# Upregulation of METTL14 mediates the elevation of *PERP* mRNA N^6^ adenosine methylation promoting the growth and metastasis of pancreatic cancer

**DOI:** 10.1186/s12943-020-01249-8

**Published:** 2020-08-25

**Authors:** Min Wang, Jun Liu, Yan Zhao, Ruizhi He, Xiaodong Xu, Xingjun Guo, Xu Li, Simiao Xu, Ji Miao, Jianpin Guo, Hang Zhang, Jun Gong, Feng Zhu, Rui Tian, Chengjian Shi, Feng Peng, Yechen Feng, Shuo Yu, Yu Xie, Jianxin Jiang, Min Li, Wenyi Wei, Chuan He, Renyi Qin

**Affiliations:** 1 Department of Biliary-Pancreatic Surgery, Affiliated Tongji Hospital, Tongji Medical College, Huazhong University of Science and Technology, 1095 Jiefang Ave, Wuhan, 430030 Hubei China; 2 Department of Chemistry, Department of Biochemistry and Molecular Biology, Institute for Biophysical Dynamics, Howard Hughes Medical Institute, The University of Chicago, Chicago, IL 60637 USA; 3 Department of Trauma Surgery, Affiliated Tongji Hospital, Tongji Medical College, Huazhong University of Science and Technology, Wuhan, 430030 China; 4 Department of Breast Surgery, The First Affiliated Hospital of Zhengzhou University, Zhengzhou, 450000 China; 5 Department of Endocrinology, Affiliated Tongji Hospital, Tongji Medical College, Huazhong University of Science and Technology, Wuhan, 430030 China; 6 Division of Endocrinology, Boston Children’s Hospital, Harvard Medical School, Boston, MA 02115 USA; 7 The First Affiliated Hospital, Sun Yat-sen University, Guangzhou, China; 8 Department of Pathology, Beth Israel Deaconess Medical Center, Harvard Medical School, 330 Brookline Avenue, Boston, MA 02215 USA; 9 Department of Hepatic-Biliary-Pancreatic Surgery, Renmin Hospital of Wuhan University, Wuhan, 430060 China; 10 Department of Medicine, The University of Oklahoma Health Sciences Center, Oklahoma City, OK USA

**Keywords:** Pancreatic cancer, *N*^6^-methyladenosine, m^6^A, METTL14, PERP

## Abstract

**Background:**

Pancreatic cancer is one of the most lethal human cancers. *N*^6^-methyladenosine (m^6^A), a common eukaryotic mRNA modification, plays critical roles in both physiological and pathological processes. However, its role in pancreatic cancer remains elusive.

**Methods:**

LC/MS was used to profile m^6^A levels in pancreatic cancer and normal tissues. Bioinformatics analysis, real-time PCR, immunohistochemistry, and western blotting were used to identify the role of m^6^A regulators in pancreatic cancer. The biological effects of methyltransferase-like 14 (METTL14), an mRNA methylase, were investigated using in vitro and in vivo models. MeRIP-Seq and RNA-Seq were used to assess the downstream targets of METTL14.

**Results:**

We found that the m^6^A levels were elevated in approximately 70% of the pancreatic cancer samples. Furthermore, we demonstrated that METTL14 is the major enzyme that modulates m^6^A methylation (frequency and site of methylation). METTL14 overexpression markedly promoted pancreatic cancer cell proliferation and migration both in vitro and in vivo*,* via direct targeting of the downstream *PERP* mRNA (p53 effector related to PMP-22) in an m^6^A-dependent manner. Methylation of the target adenosine lead to increased *PERP* mRNA turnover, thus decreasing PERP (mRNA and protein) levels in pancreatic cancer cells.

**Conclusions:**

Our data suggest that the upregulation of METTL14 leads to the decrease of PERP levels via m^6^A modification, promoting the growth and metastasis of pancreatic cancer; therefore METTL14 is a potential therapeutic target for its treatment.

## Statement of significance

Identifying the mechanisms that determine the frequency and effects of adenosine methylation (m^6^A) is essential for the rational design of new therapeutics for m^6^A-related cancers, such as pancreatic cancer. We identified METTL14 as the primary regulator of m^6^A, which suggests a new focus for targeted pancreatic cancer treatment development.

## Background

Pancreatic cancer is one of the most aggressive malignancies with a 5-year survival rate of approximately 5% [1, 2]. Genetic studies of pancreatic cancers have identified a plethora of alterations in crucial genes [3]; however, the disclosure and characterization of additional molecular mechanisms (or biomarkers) that could be considered for the development of novel therapeutic strategies for pancreatic cancer is essential. *N*^6^-methyladenosine (m^6^A), one of more than 160 mRNA nucleotide variants, has emerged as a prevalent modification in cancer [4, 5]. m^6^A-associated effects and distinct expression patterns have been reported in several types of cancer, such as glioblastoma, hepatocellular carcinoma, and leukemia. Still, the expression patterns and pathophysiological role of m^6^A in pancreatic cancer remain largely unknown. Their characterization may suggest new therapeutic strategies for pancreatic cancer [6–9].

M^6^A is detected on adenosines embedded in the consensus sequence G [G > A]m^6^AC[U > A > C] in various mRNA transcripts [10, 11]. Notably, m^6^A is a dynamic modification, induced by a methyltransferase complex comprising METTL3, METTL14, and other regulatory subunits, and removed by the RNA demethylases, FTO and ALKBH5 [10, 12, 13].

m^6^A-methylated transcripts are recognized by reader proteins that regulate different RNA processing events, such as pre-mRNA processing [14, 15], translation [16–19], and decay [19, 20]. Thus, the study of the m^6^A modification, and of the proteins that control methylation/demethylation steps, as well as of the resulting biological effects have advanced our understanding of the impact of epigenetic regulation on both physiological and pathological processes [21]. More importantly, accumulating evidence suggests that m^6^A promotes carcinogenesis [9, 14, 22, 23].

*PERP* (p53 effector related to PMP-22) is a p53 target gene involved in DNA damage-induced apoptosis by dependently or independently of p53 signal pathways [24–27]. *PERP* plays an essential role in the adhesion sub-program (affecting cell death), essential for the maintenance of epithelial integrity and homeostasis [28]. Moreover, several reports showed that *PERP* was required for oncogenic transformation, growth, apoptosis of breast cancer, and uveal melanoma cells, as a regulator of p53, p63, MKL1, and SERCA2b [29–32]. However, the effect of *PERP* in pancreatic ductal adenocarcinoma (PDAC) has not been fully elucidated.

A previous study reported that METTL3, ALKBH5 and YTHDF2 play important roles in pancreatic cancer cells [33–36]. However, the underlying mechanism by which aberrant m^6^A modifications facilitate the growth of pancreatic cancer has not been determined. To address this question, we evaluated the expression and function of m^6^A and m^6^A-associated proteins in pancreatic cancer tissues, and systematically assessed their clinical relevance using in vitro and in vivo models.

## Methods

### Cell culture, reagents and antibodies

The human pancreatic cancer cell lines PANC-1, MIA PaCa-2, and SW1990 were obtained from the American Type Culture Collection (ATCC, Manassas, VA, USA); AsPC-1, BxPC-3, Capan-2, and Panc 03.27 cells were purchased from the Cell Repository of the Chinese Academy of Sciences (Shanghai, China). The immortalized HPDE cell line was obtained from the Beijing North Carolina Chuanglian Biotechnology Research Institute (Beijing, China). Capan-2, MIA PaCa-2, and PANC − 1 cells were grown in Dulbecco Modified Eagle Medium (Gibco, Carlsbad, CA, USA) supplemented with 10% FBS (Gibco), 100 U/mL penicillin G, and 100 mg/mL streptomycin (Sigma-Aldrich, St. Louis, MO, USA). AsPC-1, BxPC-3, Panc 03.27, and HPDE were grown in 1640 medium (Gibco) supplemented with 10% FBS (Gibco), 100 U/mL penicillin G, and 100 mg/mL streptomycin (Sigma-Aldrich). All cells were grown at 37 °C in a humidified 5% CO_2_ incubator. The reagents and antibodies used in this study are listed in the Additional file 10: Table S5. Reagents and antibodies.

### Clinical samples

Surgical specimens of pancreatic cancers and matching non-tumor tissues were obtained from 39 patients (for protein and RNA extraction; the details are listed in the Additional file 11: Table S6. Sample information), and normal pancreatic specimens were obtained from 9 patients, all resected from September 2014 to December 2015. Twenty-four male and 15 female patients with pancreatic cancer were enrolled (mean age 52.3 years; range 37–66 years). All cancers were verified as adenocarcinomas. No patients received preoperative chemotherapy or radiotherapy. The use of clinical samples was approved by the Human Research Ethics Committee of the Tongji Hospital, Tongji Medical College, Huazhong University of Science and Technology (Wuhan, China), and written informed consent was obtained from all study participants. METTL3 and METTL14 levels were determined in 90 pancreatic cancer cases using pancreatic cancer tissue microarrays (TMA, OD-CT-DgPan01–007) at Outdo Biotech (Shanghai, China) and another 30 tissue sample pairs (and clinicopathological records) obtained from patients at Tongji Hospital. WTAP level was determined for 90 cancer cases using pancreatic cancer TMA (HPan-Ade180Sur-02) at Outdo Biotech (Shanghai, China).

### Expression profiling of a TCGA dataset

TCGA pancreatic cancer mRNA gene expression data and relevant clinical information were downloaded from UCSC Xena at https://xenabrowser.net/. The gene expression profile was analyzed using the Illumina HiSeq pancan normalized pattern.

### Real-time PCR and mRNA stability analysis

mRNA stability analysis was performed according to a previously described protocol [37]. Briefly, cells transfected with the indicated plasmids for 72 h were directly harvested (mRNA steady-state level) or treated with 5 mM Actinomycin D and harvested at the indicated time points. Equal RNA amounts (1 μg) were transcribed into cDNA using the PrimeScript™ RT reagent Kit (TAKARA, RR047A). Gene expression was analyzed on an ABI StepOnePlus using the SYBRGreen reagent (TAKARA, Shiga, Japan). The housekeeping gene *GAPDH* was used as the reference gene in all RT-PCR analyses. The RT-PCR primers used in this study are listed in the Additional file 10: Table S5. Reagents and antibodies.

### Western blotting

Cells were harvested and lysed in RIPA buffer with protease inhibitor cocktail for 30 min on ice. After centrifugation at 12,000 g for 15 min, the supernatants were collected as the total cellular protein extracts. Protein concentrations in lysates were determined using the bicinchoninic acid protein assay kit (Beyotime, Haimen, China). The proteins were resolved on an SDS-PAGE gel, transferred onto a polyvinylidene I fluoride membrane (Millipore, Burlington, MA, USA), and immunoblotted with the respective primary and secondary antibodies (Additional file 10: Table S5. Reagents and antibodies). The proteins were visualized using enhanced chemiluminescence.

### Gene silencing by shRNA

To generate the shRNA plasmid, fragments of shRNA targets were cloned into the AgeI-EcoRI site of pLKO.1. shRNA resistant-METTL14 plasmid was used to exclude off-target effects. Cells were transfected using Lipofectamine 2000 (Invitrogen, Carlsbad, CA, USA) as per the manufacturer’s instructions. shRNA targets are listed in the Additional file 10: Table S5. Reagents and antibodies.

### Lentivirus transfection

Lentiviral vectors harboring shCtrl (pLKO.1), shMETTL14_002, vector (pHAGE) and FLAG-METTL14 were constructed by GenePharma (Shanghai, China), and used to individually transfect cells, according to the manufacturer’s instructions. Briefly, pancreatic cancer cells were transfected for 48 h with 5 μg/mL polybrene (GenePharma, Shanghai, China). Then, cells were cultured with 5 μg/mL puromycin (Sigma-Aldrich) for 2 weeks. Selected pools of (confirmed) knockdown and overexpressing cells were used in the experiments.

### Cell viability assay

The CCK8 assay was used to evaluate cell viability. Briefly, cells were plated into a 96-well plate, at a concentration of 2000 cells per well. After adhesion, the cells were starved in serum-free medium for 12 h. Fresh complete medium with CCK-8 (1:10) was then added to each well, and the cells were incubated at 37 °C with 5% CO_2_ for 1 h. The absorbance at 450 nm was then measured using a microplate reader (ELx808, Biotek Instruments, Winooski, VT, USA).

### Colony-forming assay

A colony-forming assay was used to determine the proliferation of cells as indicated by the figures. Cells were seeded in 6-cm dishes, at a concentration of 2000 cells per dish. The medium was exchanged to fresh medium, the cells were allowed to grow for 14 days, and then stained with crystal violet (0.5% wt/vol) in PBS, and photographed to quantify the colonies formed.

### Transwell assay

Transwell inserts (24-well inserts, 8-μm pore size; Corning Inc., Corning, NY, USA) were used to determine cell invasiveness in vitro. Inserts were pre-coated with extracellular matrix gel (BD Biosciences, Bedford, MA, USA). The cells were serum-starved overnight in a serum-free medium, resuspended in a medium containing 0.1% (wt/vol) bovine serum albumin, and placed into the upper chamber of the transwell unit in triplicate. The lower chambers were filled with 10% (wt/vol) FBS as the attractant. The cells were incubated for 24 h. Then, the cells on the upper membrane surface were removed, while the cells on the lower surface were fixed in 4% (vol/vol) paraformaldehyde and stained with 0.1% (wt/vol) crystal violet solution. Stained cells were counted under a light microscope.

### Wound-healing assay

Cell monolayers (1 × 10^6^ per well) were cultured overnight in 6-well plates. After adhesion, the cell layers were scratched with a sterile plastic tip, washed two times with PBS, cultured for 24 h in a medium containing 1% (wt/vol) FBS, and imaged on a microscope.

### Immunofluorescence

Cells were incubated overnight on glass coverslips, treated as indicated, fixed in 4% (vol/vol) paraformaldehyde, and permeabilized for 20 min with 0.1% (vol/vol) Triton X-100 (Sigma-Aldrich). They were then blocked with 5% (wt/vol) bovine serum albumin for 30 min at room temperature (25 °C) and incubated overnight at 4 °C with the primary antibodies. They were then incubated with the respective fluorochrome-conjugated secondary antibodies for 1 h at 37 °C and counterstained with 4′,6-diamidino-2-phenylindole (Sigma-Aldrich) for 10 min. The cells were visualized under the confocal microscope LSM710 (Carl Zeiss, Germany).

### Immunohistochemistry

Tumor samples were embedded in paraffin and cut to a thickness of 4 μm. Sections and TMA were stained with hematoxylin and eosin, or incubated with primary antibodies (as indicated), using the ElivisionTM plus Polymer HRP immunohistochemistry kit (Maxim, Fujian, China). Images of representative fields were obtained using the Aperio ImageScope (Leica Biosystems, Wetzlar, Germany). The overall score for each section was given by the multiplication of the intensity and the positive rate scores of stained cells as previously described [38]. The staining intensity score was determined as 0 = negative, 1 = weak, 2 = moderate, and 3 = strong. The positive rate score was determined as 0 = negative, 1 = (1–25%), 2 = (26–50%), 3 = (51–75%) and 4 = (76–100%). IHC scores superior to 6 in cancer tissues were defined as “high expression”.

### m^6^A colorimetric quantification

Total RNA was extracted from pancreatic cancer cells and tissues using TRIzol (ref.15596–018; Invitrogen, Carlsbad, CA, USA) and treated with DNase I (ref.11284932001; Sigma-Aldrich) as per the manufacturers’ instructions. RNA samples were analyzed using a NanoDrop ND-2000 spectrophotometer (NanoDrop Tech). m^6^A levels in total RNA were evaluated using the EpiQuik™ m^6^A RNA methylation quantification kit (ref.P-9005; EpiGentek, Farmingdale, NY, USA) according to the manufacturer’s instructions.

### m^6^A high-performance liquid chromatography/mass spectrometry (HPLC/MS) quantification

Total RNA was isolated from pancreatic cancer cells and tissues using TRIzol (Invitrogen) as per the manufacturer’s instructions, and treated with DNase I (Sigma). Polyadenylated RNA was enriched from total RNA using the GenElute mRNA miniPREP kit (ref. MRN70; Sigma-Aldrich). Nucleosides were analyzed using an LC-ESI-MS/MS as reported elsewhere [39]. The RNA m^6^A content was acquired and processed using the ABSCIEX Analyst 1.5 software (Applied Biosystems, Foster City, CA, USA). HPLC separation was performed using an Hisep C18-T column (150 mm, 2.1 mm inner diameter, 5 μm; Weltech Co, Ltd., Gyeonggi-do, Korea) with a flow rate of 0.2 mL/min at 35 °C. Formic acid in water [0.1%, (vol/vol), solvent A)] and a mixture of 0.1% formic acid in methanol [solvent B (vol/vol)] were used as the mobile phase. A gradient of 5 min of 5% B, 10 min of 5–30% B, 5 min of 30–50% B, 3 min of 50–5% B, and 17 min of 5% B was used. m^6^A levels superior to the average value (0.231%) in cancer tissues were defined as “high”; those inferior to the average value in cancer tissues were defined as “low”.

### Pancreatic Cancer models in Balb/C nude mice

Animal experiments were approved by the Institutional Animal Care and Treatment Committee of Huazhong University of Science and Technology. Female nude BALB/c mice (6–8 weeks old) were obtained from HFK BioTechnology.

For the subcutaneous transplantation model, 100 μL of 1 × 10^6^ cells were injected subcutaneously into the right armpit of BALB/c nude mice. Animal weight and tumor diameter were measured once a week from the time of implantation.

For the pancreatic cancer orthotopic implantation model, 200 μL of Panc02-lucifer cells (2 × 10^7^) were injected into the pancreas in mice anesthetized and laparotomized. After 4 weeks, the mice were anesthetized and injected with 150 mg/kg d-luciferin, via the tail vein. Mice were then placed into the imaging chamber of the IVIS Lumina XR apparatus (PerkinElmer, Waltham, MA, USA), and white-light and bioluminescence images were acquired.

For the liver metastasis model, BALB/c nude mice received 2 × 10^6^ cells (in 100 μL DMEM), directly injected into the spleen. Their body weight was measured once a week from the time of implantation. Survival was recorded. At the experimental endpoints, liver tissues were harvested, imaged, embedded in 10% paraffin, and subjected to immunohistochemical staining.

### MeRIP-Seq and MeRIP qPCR

MeRIP-Seq was performed as previously described [18, 19]. Briefly, poly-A–purified RNA was fragmented and incubated with an anti-m^6^A antibody. The mixture was immunoprecipitated via incubation with protein A beads (Thermo Fisher Scientific, Waltham, MA, USA). The captured RNA was washed and purified with the RNA clean and concentrator kit (Zymo Research, Tustin, CA, USA). Total mRNA and 200 ng of immunoprecipitated RNA from each sample were sequenced and used for library construction using the Illumina Hiseq 2000 platform, as per the manufacturer’s instructions. m^6^A-seq data were analyzed according to a protocol previously described [40]. In brief, Tophat2 (version 2.2.1) with Bowtie1 support was used to align the sequence reads to the reference genome and transcriptome (hg19) [41]. Then the exomePeak R/Bioconductor package (version 3.7) was used to find m^6^A peaks. Significant peaks with false discovery rates (FDR) lower than 0.05 were annotated to the RefSeq database (hg19). Sequence motifs were identified using the Homer software (version 4.9) [42], and the DAVID analysis tool (version 6.8) was used to perform GO term enrichment analysis [43]. For MeRIP qPCR, briefly, the precipitated product was reverse-transcribed and analyzed by PCR. The primers used are listed in the Additional file 10: Table S5. Reagents and antibodies.

### RNA-Seq

Total RNA was extracted using the TRIzol reagent (Invitrogen), according to the manufacturer’s protocol. RNA was sequenced at BGI (Beijing Genomics Institute) using the BGISEQ-500 platform. Briefly, mRNA was enriched by oligo-dT selection or rRNA depletion. Subsequently, it was purified, fragmented, and reverse-transcribed into cDNA, which was then end-repaired and 3′-adenylated. This was followed by adaptor ligation. Ligation products were purified and PCR-amplified, to enrich the purified cDNA templates, using PCR primer fragments. PCR products were then heat-denatured, and ssDNA was cyclized by splint-oligo and DNA ligase. Finally, the prepared library was sequenced.

### Plasmid construction and luciferase reporter assay

To generate wild-type or mutated pmiR-RB-Report-PERP-3′-UTR plasmids, the appropriate 3′-UTR (0-1000 nt) fragments were cloned into a pmiR-RB-Report plasmid. The 3′-UTR mutated sequence was constructed via the substitution of an A to a T at position 808. The cloned sequences are listed in the Additional file 10: Table S5. Reagents and antibodies. Luciferase activity was determined using the dual-luciferase reporter assay system (Promega, Madison, WI, USA), as per the manufacturer’s instructions. Relative luciferase activity was determined using a GloMax 20/20 Luminometer (Promega). Luciferase activity was normalized to that of firefly luciferase. To construct PERP overexpression (CDS and 3′-UTR) plasmids, wild type or the A808T mutated sequence were cloned into the pHAGE plasmid.

### Statistical analysis

Statistical analyses were performed using the SPSS 13.0 (SPSS, Chicago, IL, USA) or Prism 5.0 (GraphPad Software, La Jolla, CA, USA) software. Data are presented as the mean ± SD or mean ± SEM of at least three independent experiments unless otherwise indicated. Significance levels were evaluated using the two-tailed Student’s *t*-test (for comparison between two groups) or the one-way ANOVA (for comparisons of more than 2 groups). *p* < 0.05 was considered statistically significant.

## Results

### m^6^A modification levels are elevated in pancreatic Cancer

**Fig. 1:**
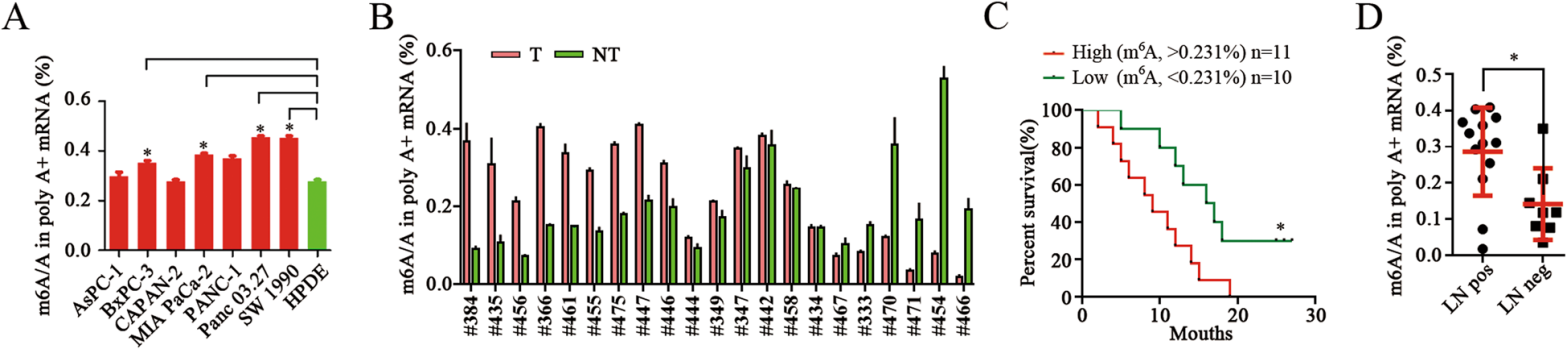
m^6^A modification levels and profiles in pancreatic cancer. **a**, **b** HPLC quantification of m^6^A levels in mRNA extracted from pancreatic cancer cell lines and human pancreatic cancer tissues, as indicated. * *p* < 0.05. **c** Kaplan-Meier analysis of the correlation between the m^6^A levels and the overall survival of pancreatic cancer patients. * *p* < 0.05. **d** Quantification of m^6^A levels in 21 pancreatic cancer tissues with (LN Pos) or without (LN Neg) lymphatic metastasis. * *p* < 0.05

We measured m^6^A levels in pancreatic cancer cell lines and human pancreatic cancer tissue samples. Notably, m^6^A levels were elevated in five of seven pancreatic cancer cell lines compared to human pancreatic ductal epithelial (HPDE) cells and normal pancreatic tissues (Fig. 1a; Additional file 1: Fig. S1A). Similarly, m^6^A levels were higher in approximately 70% of pancreatic cancer tissues, compared to those in pair-matched adjacent tissues (Fig. 1b; Additional file 1: Fig. S1B). Furthermore, the relationship between m^6^A levels and clinicopathology was analyzed (Additional file 6: Table S1). Poor overall survival was significantly correlated with higher levels of m^6^A (Fig. 1c), and patients with tumors expressing higher m^6^A levels developed significantly more lymphatic metastases than patients with tumors expressing lower levels (Fig. 1d).

Next, we performed *N*^6^-methyladenosine-sequencing (m^6^A-seq), using pair-matched pancreatic tumor and adjacent tissue samples from one patient with pancreatic cancer, and one normal pancreatic tissue sample (N) from a patient with pancreatic trauma (Additional file 7: Table S2). We observed that m^6^A peaks were enriched near the start and stop codons and were characterized by the canonical GGACU motif in all samples (Additional file 1: Figs. S1D, S1E). Then we analyzed the unique m^6^A peaks and transcripts comparing tumor and adjacent tissues (T vs. S), and tumor and normal tissues (T vs. N) (Additional file 1: Fig. S1C). Most unique peaks were distributed in the exon, the 3′-untranslated region (UTR), and introns; a few unique peaks were mapped to the 5′-UTR (Additional file 1: Fig. S1F). To explore the m^6^A peaks specific to pancreatic cancer, we analyzed the m^6^A peaks and gene coding transcripts in cancer tissue (T), adjacent tissue (S), and normal pancreatic tissue (N) samples. Gene ontology (GO) analysis demonstrated that unique m^6^A-modified transcripts were mainly involved in metabolic processes, cell connection, and kinase activity (Additional file 1: Fig. S1G). The KEGG pathway analysis demonstrated that unique m^6^A-modified transcripts were associated with mRNA Splicing, p53 effectors, interferon α/β, TGF-β and Rho GTPases (Additional file 1: Fig. S1H). These results suggest that pancreatic cancer tissues have distinct m^6^A profiles that differentiate them from normal tissues.

### Aberrant expression of METTL14 in pancreatic Cancer

**Fig. 2:**
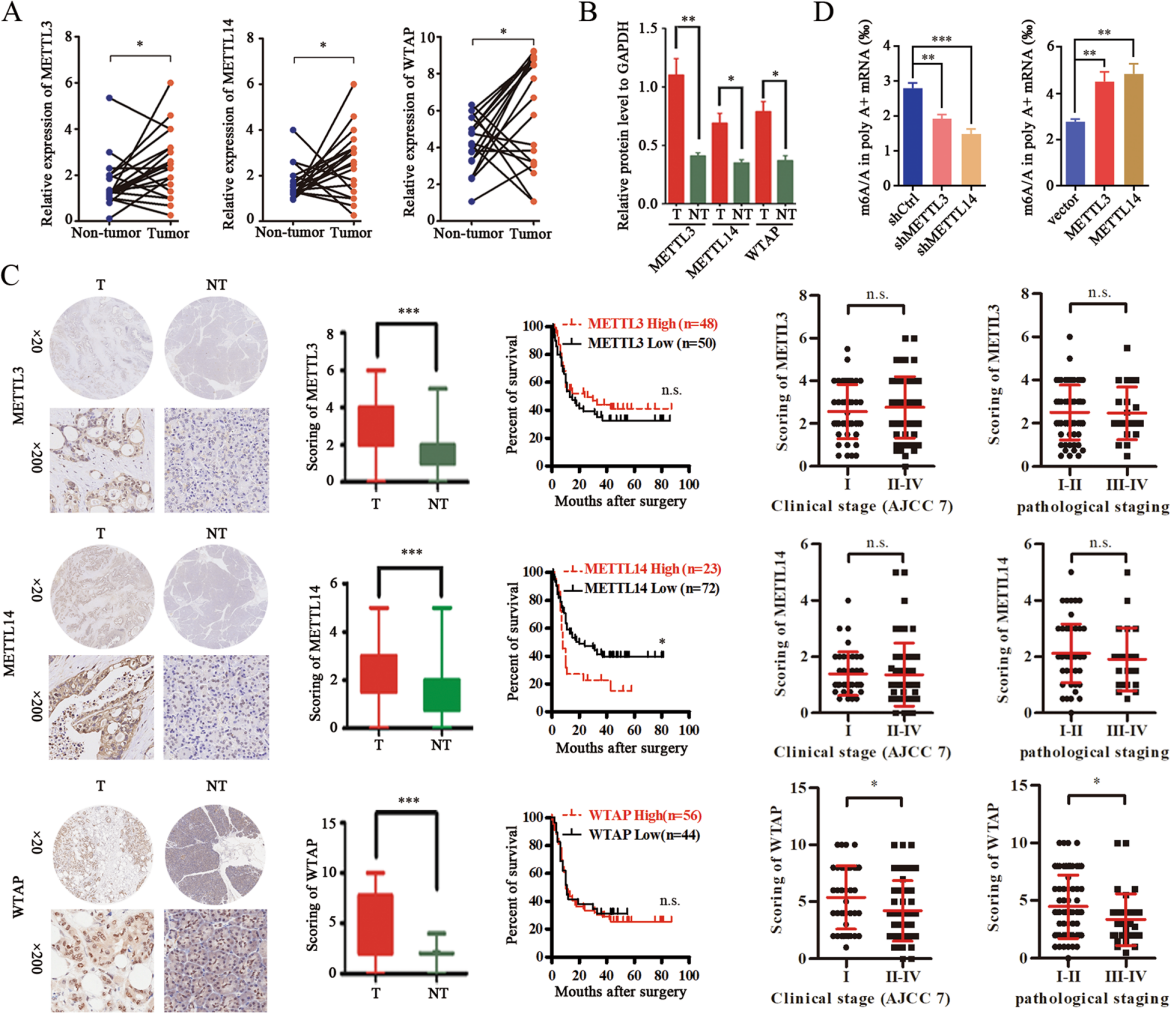
Aberrant expression of METTL 3-METTL14 complex in pancreatic cancer. **a** Real-time PCR analysis of the relative mRNA levels of *METTL3*, *METTL14*, and *WTAP* in 20 paired pancreatic cancer tissues. * *p* < 0.05. **b** Quantification of western blot analyses of METTL3, METTL14, and WTAP levels in pancreatic cancer and adjacent tissues. * *p* < 0.05; ** *p* < 0.01. **c** Immunohistochemical analysis of METTL3/METTL14/WTAP expression in pancreatic cancer tissues; Kaplan-Meier analysis of the correlation between METTL3/METTL14/WTAP expression and the overall survival of pancreatic cancer patients; and relative METTL3/METTL14/WTAP expression scores in pancreatic cancer tissues per clinical and pathological stage. * *p* < 0.05; ***p < 0.001; n.s., no significance. **d** Colorimetric quantification of m^6^A in PANC-1 cells after *METTL3* and *METTL14* knockdown or overexpression. ** *p* < 0.01; ***p < 0.001

To elucidate the molecular mechanisms responsible for elevated m^6^A levels in pancreatic cancer, we assessed the expression of the most important m^6^A regulatory factors (METTL3, METTL14, and WTAP, which form a complex) in the paired cancer and adjacent tissue samples. Notably, real-time PCR revealed that *METTL3*, *METTL14,* and *WTAP* were upregulated in pancreatic cancer tissues compared with adjacent, healthy tissues (Fig. 2a). Western blot also showed that METTL3 METTL14 and WTAP levels were significantly higher in pancreatic cancer samples compared with normal tissue samples. (Fig. 2b; Additional file 2: Fig. S2). However, among the complex components, only METTL14 levels were significantly associated with patient survival (Fig. 2c): elevated METTL14 levels were associated with poor overall survival (Fig. 2c). Together, these data suggest that METTL14 is a major m^6^A regulating factor, involved in the clinicopathology of pancreatic cancer.

### METTL14 Upregulation promotes pancreatic Cancer growth and metastasis

**Fig. 3:**
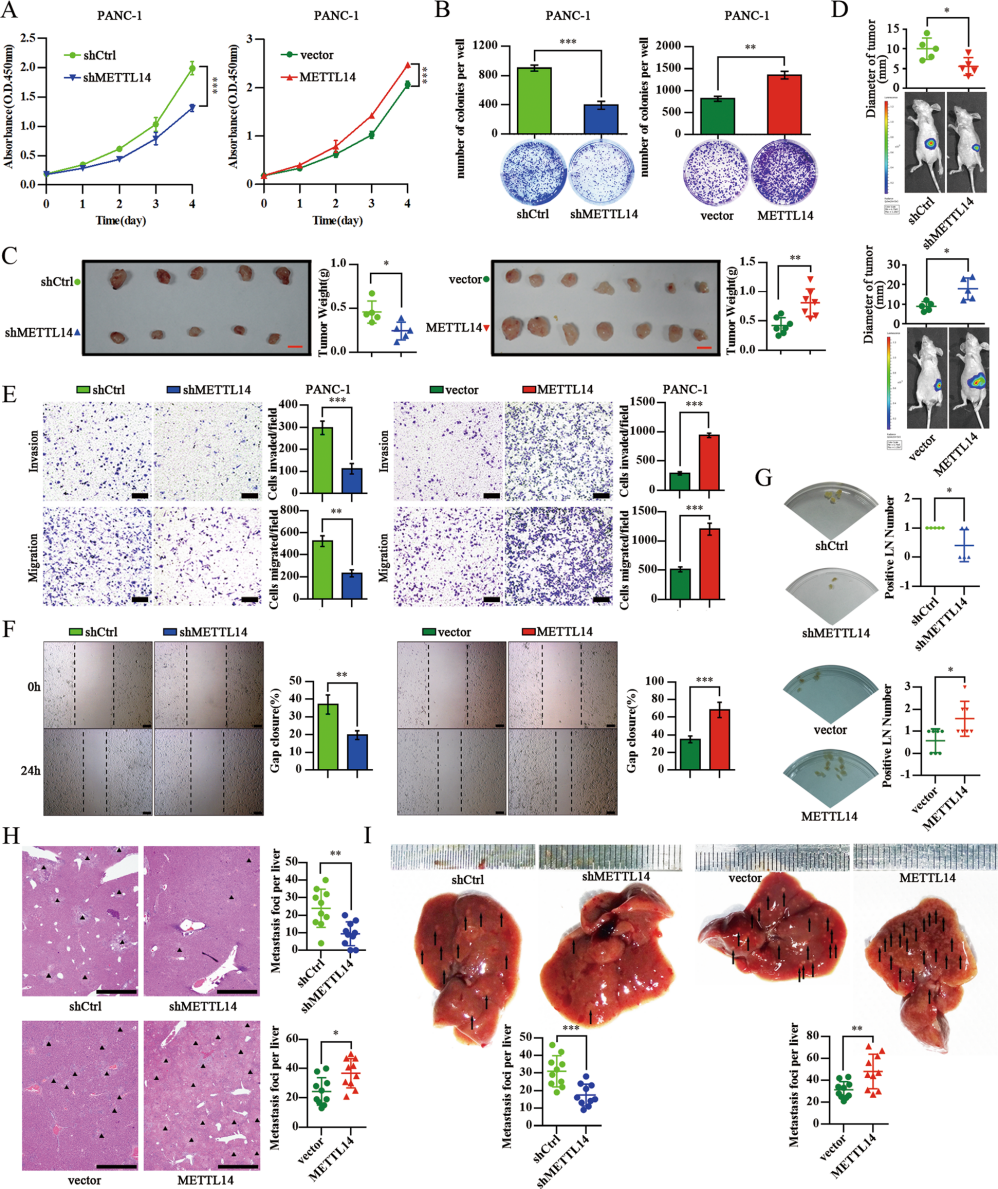
Upregulation of METTL14 enhances the growth and metastasis of pancreatic cancer. **a** Viability of PANC-1 cells expressing shCtrl, shMETTL14, vector or exogenous METTL14 detected by the CCK8 assay. ***p < 0.001. **b** Representative images from the colony-forming assay (lower panel) and colony number analysis (upper panel). All experiments were performed in triplicate and data are presented as the mean ± SD. **p < 0.01; ***p < 0.001. **c** Images (left panel; scale bar: 1 cm) and weight analysis (right panel) of subcutaneous tumors from the indicated groups. * *p* < 0.05; ** *p* < 0.01. **d** Images of the orthotopic transplantation mouse model (shCtrl, shMETTL14, vector or METTL14 groups; lower panel), and analysis of the orthotopic tumor diameter (upper panel). * *p* < 0.05. PANC-1 cells expressing shCtrl, shMETTL14, vector or METTL14 were subjected to a transwell assay with or without Matrigel (Scale bar: 200 μm) (**e**), and to a wound-healing assay (Scale bar: 200 μm) (**f**). All experiments were performed in triplicate and data are presented as the mean ± SD. ** *p* < 0.01; ***p < 0.001. **g** Images of armpit lymph node metastasis in the subcutaneous implantation model (left panel) and the respective quantitative analysis (right panel). * *p* < 0.05. **h** Statistical analysis of the average number of liver metastases per group in the orthotopic transplantation mouse model. Scale bar: 1 mm. * *p* < 0.05; ** *p* < 0.01. **i** Statistical analysis of the average number of liver metastases per group in the liver metastasis model. ** *p* < 0.01; ***p < 0.001

To assess the biological role of METTL14 in pancreatic cancer, we overexpressed or knocked down *METTL14* in human pancreatic cancer cell lines (Additional file 3: Figs. S3A-D). Consistently with its documented catalytic role in m^6^A methylation, depletion of *METTL14* markedly diminished m^6^A levels (Fig. 2d). *METTL14* knockdown significantly suppressed the proliferation and colony formation of PANC-1 and MIA PaCa-2 cells, whereas, ectopic expression of *METTL14* increased the proliferation and colony formation of PANC-1 and BxPC-3 cells (Fig. 3a, b; Additional file 3: Figs. S3E-G). Notably, we observed that elevated METTL14 expression enhanced tumor growth in both subcutaneous and orthotopic transplantation models in nude mice. Conversely, depletion of *METTL14* effectively suppressed tumor growth in these models (Fig. 3c, d; Additional file 3: Fig. S3H). These observations suggest that METTL14 promotes the growth of pancreatic cancer in vitro and in vivo.

Next, we examined the role of METTL14 in invasion and metastasis in the context of pancreatic cancer. To this end, cell migration assays revealed that *METTL14* depletion reduced the migration and invasiveness of PANC-1 and MIA PaCa-2 cells, whereas overexpression of *METTL14* exerted the opposite effect on PANC-1 and BXPC-3 cells (Fig. 3e; Additional file 3: Figs. S3I, S3J). Similar migration data were obtained in a wound-healing assay (Fig. 3f; Additional file 3: Fig. S3K). We further explored metastasis in vivo using three mouse models. In a subcutaneous implantation model, we observed that *METTL14* depletion or overexpression significantly decreased or increased lymphatic metastases, respectively (Fig. 3g). In an orthotopic transplantation model, *METTL14* overexpression significantly accelerated pancreatic cell metastases to the liver, while *METTL14* depletion reduced liver metastases (Fig. 3h). Furthermore, the overexpression of *METTL14* led to a significant increase in liver metastases and reduced the overall survival, while *METTL14* depletion decreased the number of micro-metastases and prolonged survival in a mouse model of liver metastasis (Fig. 3i; Additional file 3: Fig. S3L). Together, these data indicate that METTL14 plays an important role as a promotor of pancreatic cancer growth and metastasis.

### Identification of METTL14 downstream targets by RNA-Seq and m^6^A-Seq

**Fig. 4:**
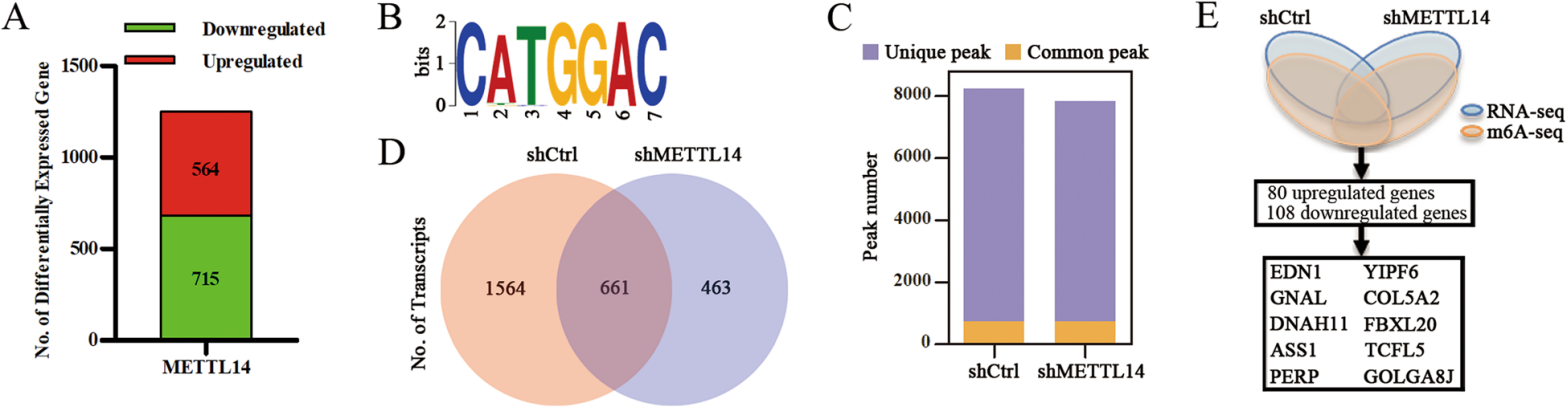
Identification of METTL14 targets via RNA-Seq and m^6^A-Seq. **a** Differentially expressed genes with over 2-fold expression changes in PANC-1 cells treated with shMETTL14 compared with those treated with shCtrl. **b** Top consensus motif identified from m^6^A-Seq peaks in PANC-1-shCtrl and PANC-1-shMETTL14 cells. **c**, **d** Number of m^6^A peaks and m^6^A-modified transcripts identified from m^6^A-Seq peaks in PANC-1-shCtrl and PANC-1-shMETTL14 cells. **e** Schematic diagram of METTL14 downstream analysis

To investigate the regulatory role of METTL14 in pancreatic cancers, we performed RNA-Seq to analyze the gene expression profiles of PANC-1 cells, control or *METTL14* deficient. We observed that 564 genes were upregulated and 715 genes were downregulated after *METTL14* knockdown (Fig. 4a; Additional file 8: Table S3). GO analysis revealed that the differentially expressed genes were significantly enriched in gene sets associated with cellular processes, metabolism, protein binding, and catalysis (Additional file 4: Fig. S4A). Furthermore, the KEGG pathway analysis revealed that the largest subset of differentially expressed genes was associated with pancreatic cancer, and with the VEGF, mTOR, and insulin signaling pathways (Additional file 4: Figs. S4B, S4C).

Next, we used m^6^A-Seq to map the m^6^A methylomes in PANC-1 cells with physiological (shCtrl) and reduced (shMETTL14) *METTL14* levels. Consistently with our previous data, the GGACU motif was highly enriched in m^6^A sites in both control and *METTL14* knockdown cells (Fig. 4b). We identified 8238 and 7820 m^6^A peaks derived from 2225 and 1124 m^6^A-modified transcripts, of which 7496 and 7078 peaks derived from 1564 and 463 transcripts were unique in the control and *METTL14* knockdown cells, respectively (with 742 common peaks and 661 transcripts) (Fig. 4c, d; Additional file 4: Fig. S4D; Additional file 9: Table S4). GO analysis of unique transcripts revealed that the differentially expressed genes were significantly enriched in gene sets associated with cytoskeletal protein binding, GTPase regulation, and cell projection organization (Additional file 4: Fig. S4E). Furthermore, the KEGG pathway analysis demonstrated that some peaks were associated with Rap1 and signaling pathways that regulate the pluripotency of stem cells (Additional file 4: Fig. S4F). To assess whether altered gene expression was a consequence of METTL14-mediated methylation (particularly m^6^A), we compared the data derived from RNA-Seq and m^6^A-Seq. RNA-Seq identified 80 upregulated genes and 108 downregulated genes showing m^6^A modifications, including the top six genes whose levels were increased: *EDN1*, *GNAL*, *DNAH11*, *ASS1*, *PERP*, and *YIPF6* (Fig. 4e).

### *PERP* is an essential METTL14 target gene in pancreatic Cancer

**Fig. 5:**
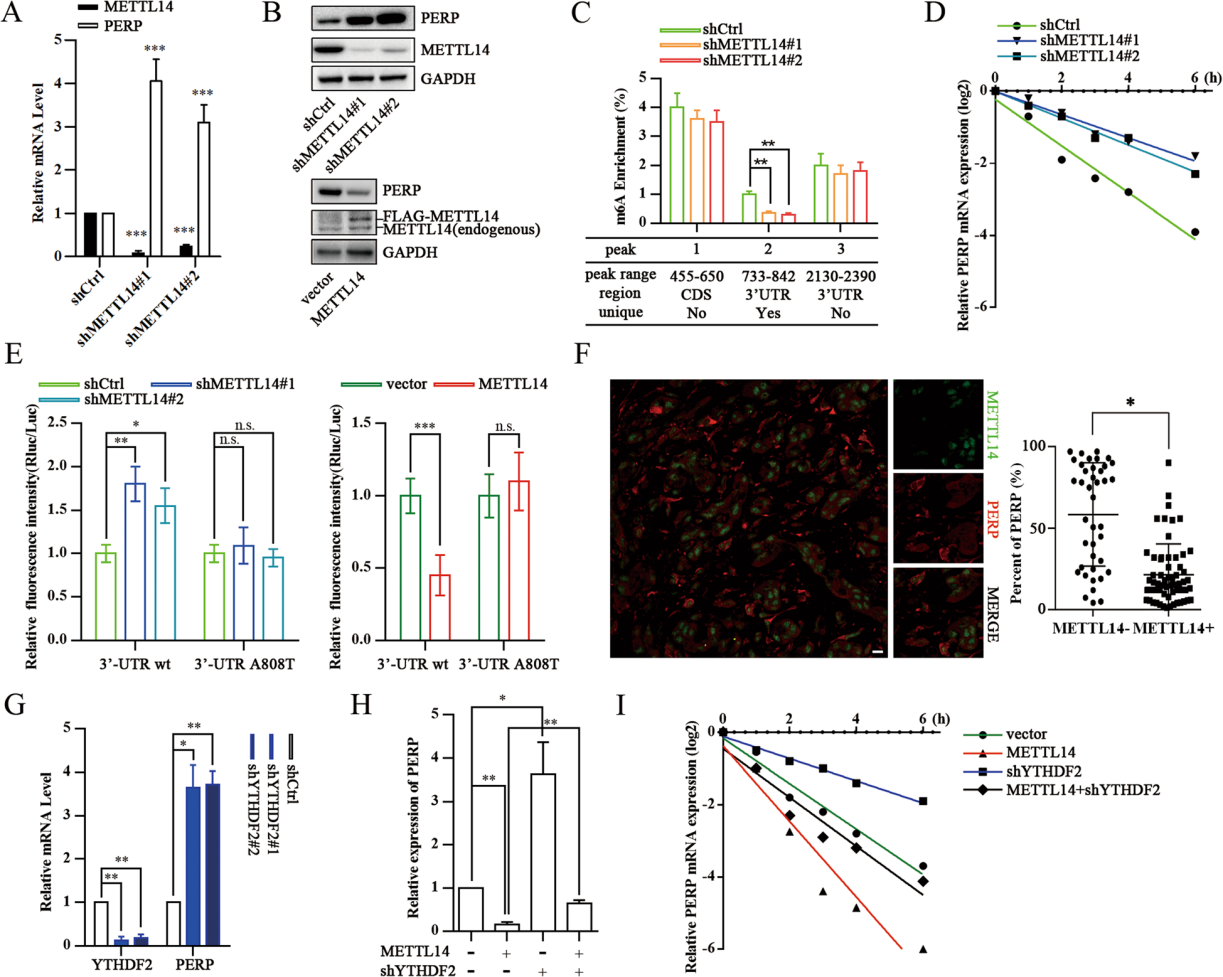
PERP is the key target of METTL14 in pancreatic cancer. **a** qPCR analysis of *METTL14* and *PERP* in PANC-1 cells expressing shCtrl or shMETTL14. ***p < 0.001. **b** Western blotting of METTL14 and PERP in PANC-1 cells expressing shCtrl, shMETTL14, vector or METTL14. **c** MeRIP-qPCR analysis of fragmented *PERP* RNA from PANC-1 (control and *METTL14* depleted) cells. ** *p* < 0.01. **d** qPCR analysis of *PERP* mRNA levels in PANC-1 cells (control and *METTL14* depleted) after actinomycin D treatment. **e** PANC-1 cells were pre-transfected with wild-type or mutated pmiR-RB-Report-PERP-3′UTR plasmids, and then treated as indicated. Renilla luciferase activity was normalized to firefly luciferase activity and expressed as the mean ± SD. * *p* < 0.05; ** *p* < 0.01; ***p < 0.001; n.s., no significance. **f** Correlation between METTL14 and PERP protein levels in pancreatic cancer specimens. Left - representative IF images of pancreatic cancer specimens. Scale bar, 20 μm. Right - percentage of PERP-positive cells among METTL14-positive versus METTL14-negative cells in selected microscope fields of each tumor (compared by the *t*-test). * *p* < 0.05. **g** qPCR analysis of *PERP* in PANC-1 cells (control and *YTHDF2* depleted). (H) qPCR analysis of *PERP* in PANC-1 cells (control and *YTHDF2* depleted) in the absence or presence of METTL14 overexpression. * *p* < 0.05; ** *p* < 0.01. **i** qPCR analysis of *PERP* mRNA levels in PANC-1 cells (control and *YTHDF2* depleted) in the absence or presence of METTL14 overexpression, and after actinomycin D treatment

To further investigate the METTL14 target genes, we validated the expression of the six most upregulated genes identified by RNA-Seq and m^6^A-Seq in *METTL14* depleted PANC-1 cells (Additional file 5: Fig. S5A). Among these targets, *PERP* mRNA and protein levels increased upon *METTL14* depletion (Fig. 5a, b). To confirm that *PERP* mRNA undergoes METTL14-mediated m^6^A modification, as determined by m^6^A-Seq, we performed methylated RNA immunoprecipitation quantitative PCR (MeRIP-PCR). These results also indicated that METTL14 could methylate *PERP* mRNA (Fig. 5c). Knockdown of *METTL14* led to a marked increase in the *PERP* transcript half-life (from 1.19 to 2.87 h) after treatment with the transcriptional inhibitor actinomycin D (Fig. 5d). Analyzing our m^6^A-Seq data derived from shMETTL14 cells as well as additional information retrieved from three independent m^6^A databases (SRAMP, RMBase, and m^6^Avar), we identified one unique peak in the 3′-UTR of *PERP* as a potential target of METTL14. Using a *PERP* 3′-UTR-reporter luciferase assay we found that knockdown of *METTL14* largely increased the luciferase activity of constructs harboring the wild type *PERP* 3′-UTR, and overexpression of *METTL14* significantly reduced the luciferase activity of constructs harboring the wild type *PERP* 3′-UTR. However, either knockdown or overexpression of *METTL14* did not alter the luciferase activity of constructs harboring the mutated *PERP* 3′-UTR sequence (Fig. 5e). To further disclose a potential correlation between PERP and METTL14, we analyzed a TCGA dataset containing PERP and METTL14 mRNA expression data [44]. The expression of PERP mRNA was negatively associated with METTL14 mRNA expression, and there was a statistically significant difference between PERP and METTL14 expression (Additional file 5: Fig. S5B). Similar results were obtained when we correlated PERP mRNA and METTL14 protein expression levels of the 20 pairs of specimens studied (Additional file 5: Fig. S5C). To further examine the association between PERP and METTL14 expression, we performed immunofluorescence assays in pancreatic cancer tissues, and observed that tumor cells with high METTL14 expression showed low PERP expression, and vice versa (Fig. 5f).

Furthermore, as the first characterized readers of m^6^A, YT521-B homology domain family (YTH) proteins regulate mRNA stability and translation [9, 18, 19]. To ascertain whether YTHDF2 is a potential reader of *PERP* m^6^A methylation, we knocked down *YTHDF2*, and observed a strongly augmented *PERP* expression in pancreatic cells (Fig. 5g). *YTHDF2* knockdown not only increased the levels and stability of *PERP* mRNA, but also abrogated their decrease under METTL14 overexpression (Fig. 5h, i). These results demonstrate that PERP is a direct target of METTL14, in an m^6^A-dependent manner that regulates the METTL14-YTHDF2-PERP axis.

### PERP is responsible for the METTL14-induced pancreatic Cancer cells’ growth and invasion

**Fig. 6:**
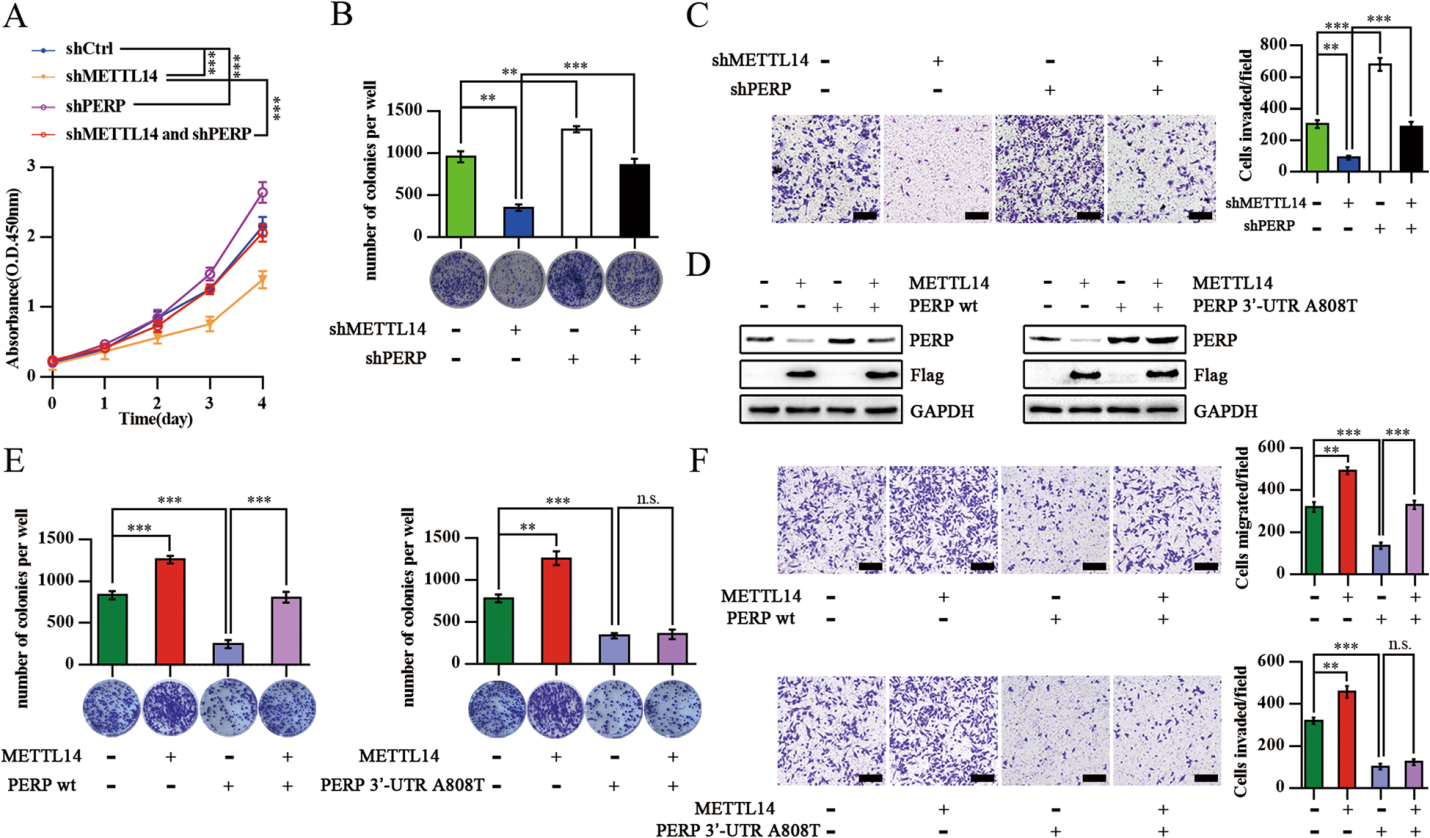
PERP is involved in the METTL14-induced Pancreatic Cancer Cells’ Growth and Invasion. **a** Viability of PANC-1 cells with or without *PERP* knockdown in the absence or presence of *METTL14* knockdown analyzed by the CCK8 assay. ***p < 0.001. **b** Representative images from the colony-forming assay (lower panel) and colony number analysis (upper panel) as indicated. All experiments were performed in triplicate and data are presented as the mean ± SD. **, *p* < 0.01; ***, *p* < 0.001. **c** PANC-1 cells with or without *PERP* knockdown in the absence or presence of *METTL14* knockdown were analyzed in a transwell assay with Matrigel. All experiments were performed in triplicate and data are presented as the mean ± SD. Scale bar: 200 μm. ** *p* < 0.01; ***p < 0.001. **d** Western blotting of PERP and Flag in PANC-1 cells with or without *PERP* WT transfection in the absence or presence of *METTL14* overexpression (left panel); western blotting of PERP and Flag in PANC-1 cells with or without *PERP* 3′-UTR transfection in the absence or presence of *METTL14* overexpression (right panel). **e** Colony-forming assay in PANC-1 cells with or without *PERP* WT transfection in the absence or presence of *METTL14* overexpression (left panel); colony-forming assay in PANC-1 cells with or without *PERP* 3′-UTR transfection in the absence or presence of *METTL14* overexpression (right panel). ** *p* < 0.01; ***p < 0.001; n.s., no significance. **f** Transwell assay in PANC-1 cells with or without *PERP* WT transfection in the absence or presence of *METTL14* overexpression (upper panel); transwell assay in PANC-1 cells with or without *PERP* 3′-UTR transfection in the absence or presence of *METTL14* overexpression (lower panel). Scale bar: 200 μm. ** *p* < 0.01; ***p < 0.001; n.s., no significance

To understand the role of PERP in METTL14-induced pancreatic cancer growth, we knocked down *PERP* in pancreatic cancer cells depleted of *METTL14*. We found that *PERP* depletion notably increased the viability and colony formation of PANC-1 cells, but also abrogated the decrease of it under knockdown of METTL14 (Fig. 6a, b). Furthermore, the transwell assay revealed that *PERP* knockdown also significantly counteracted the *METTL14* depletion-dependent inhibition of pancreatic cancer cells invasion ability (Fig. 6c). In addition, we constructed plasmids coding for PERP WT or PERP with a specific 3′-UTR site mutation (that does not prevent PERP expression) and evaluated their impact (after transfection) on the tumorigenic properties of pancreatic cancer cells overexpressing METTL14. We observed that METTL14 overexpression decreased the PERP expression levels and increased the colony formation and invasive abilities of pancreatic cancer cells, treated or not with the construct designed for PERP WT overexpression (Figs. 6d-f). However, METTL14 overexpression did not impact cancer cells overexpressing PERP with a 3′-UTR mutation (Figs. 6d-f). These findings suggest that PERP is the major effector through which METTL14 promotes the growth of pancreatic cancer.

## Discussion

Pancreatic cancer is a devastating disease associated with a complex and still not completely understood physiopathology [1, 2]. Although previous studies have identified crucial gene alterations in pancreatic cancer, effective treatments are still not available [3, 45, 46]. Recent studies have confirmed that abnormal epigenetic regulation of gene function, e.g. via *N*^6^-methyladenosine (m^6^A) modifications, plays an important role in cancer progression [21, 47]. In this study, we demonstrate that m^6^A modification levels are elevated in pancreatic cancer. We then show that the dysregulation of METTL14 can affect m^6^A levels in pancreatic cancer cells. We further provide evidence that METTL14 promotes the growth and metastasis of pancreatic cancer, and identify *PERP* as an important METTL14 target gene. Overall, mechanistically, METTL14 dysregulation leads to increased m^6^A modifications in the PERP 3′-UTR, promoting the growth and metastasis of pancreatic cancer. These observations reveal a new layer of epigenetic alterations that contribute to the development of pancreatic cancer and provide new and promising targets for the development of novel interventional therapies.

The tightly regulated m^6^A modifications play an extremely important role in the maintenance of multiple biological activities [4, 5, 16, 17, 20]. Several studies have demonstrated the involvement of dysregulated m^6^A in many human diseases, including cancers [21, 47]. In fact, m^6^A dysregulation occurs in several types of cancer and can affect key tumor suppressor and oncogene signaling pathways (and cancer progression), via alteration of RNA stability and RNA translation efficiency [5, 7, 17, 21]. Serving as the key methyltransferase responsible for m^6^A modifications, METTL14 was demonstrated to suppress the metastatic potential of hepatocellular carcinoma via m^6^A-dependent primary microRNA processing events [23]. However, little is known about the distinct expression patterns of these regulators, particularly METTL14, or their precise tumorigenic contributions for various malignancies, including pancreatic cancer [47].

In pancreatic cancer, it was reported that METTL3 promotes cancer progression and chemo- and radio-resistance [34, 48]. Although it was confirmed that ALKBH5 functions as a tumor-suppressor gene, involved in sensitizing pancreatic cancer cells to chemotherapy via direct impact on Wnt inhibitory factor 1, the m^6^A eraser was also reported to prevent pancreatic cancer progression by posttranscriptional activation of PER1 in an m^6^A-YTHDF2-dependent Manner [35, 36]. Here, we present the first study on the expression of METTL14, one of the main m^6^A regulators, in pancreatic cancer. We show for the first time that METTL14 functions as an oncogene, promoting the growth and metastasis of pancreatic cancer.

It was reported that m^6^A affects RNA expression in different ways, depending on the m^6^A modified RNA target/reader [49]. For example, YTHDF1 is generally considered to promote protein synthesis via interactions with the translation machinery, whereas YTHDF2 is believed to increase the degradation of mRNA via the reduction of the stability of target transcripts [17]. According to the results of the present study, we observed that 564 genes were upregulated and 715 genes were downregulated after *METTL14* knockdown; moreover we identified 8238 and 7820 m^6^A peaks derived from 2225 and 1124 m^6^A-modified transcripts, of which 7496 and 7078 peaks derived from 1564 and 463 transcripts were unique in the control and *METTL14* knockdown cells, respectively (with 742 common peaks and 661 transcripts). In this study, we further highlight the importance of abnormal mRNA methylation-related gene expression (and the consequent biological functions), particularly in the context of *METTL14* knockdown in human pancreatic cancer cells. Of note, we focused on particular genes whose mRNA levels were different after m^6^A modification. However, we need to keep in mind that genes with no change in mRNA levels may also play an important role in pancreatic cancer (e.g. through the different readers).

In this study, we further found that *PERP* is an essential METTL14 target gene in pancreatic cancer, obviously in an m^6^A-dependent manner. PERP is a tetraspan plasma membrane (PM) protein involved in cell-cell adhesion and in the regulation of apoptosis in many cell types [24, 27, 28, 30]. PERP positively influences its own expression and mediates apoptosis via both the extrinsic and mitochondrial pathways, dependently or independently of p53 [32, 50]. PERP was concomitantly independently identified as a protein that was downregulated in several human cancers, suggesting that PERP acts as a tumor suppressor [29, 30]. Importantly, the multi-faceted role of PERP in cancer involves well-documented functions in the mediation of apoptosis and cell-cell adhesion, epithelial-mesenchymal transition, and crosstalk with inflammation signaling pathways via interaction with *p63*, *p53*, *MKL1* and *SERCA2b* [27, 28, 32, 50]. In line with the abovementioned, we found that PERP inhibits the proliferation and metastasis of pancreatic cancer cells. Importantly, since PERP is the major effector through which METTL14 promotes the growth of pancreatic cancer, we suggest it as a potential therapeutic target. Additionally, also METTL14 should be considered, for the development of novel drugs targeting pancreatic cancer.

In summary, our study revealed elevated levels of m^6^A methylation in pancreatic cancer caused by the dysregulation of METTL14, an m^6^A modulator. We also demonstrated the critical role of METTL14 in the growth and metastasis of pancreatic cancer via targeting of *PERP* mRNA. The current study not only provides novel insights into the molecular mechanisms underlying the pancreatic cancer pathogenesis but also paves the way for the development of more effective therapeutic strategies for pancreatic cancer, targeting m^6^A regulators.

## Supplementary information

**Additional file 1: Figure S1**. m^6^A modification levels and profile in pancreatic cancer. (A, B) Colorimetric quantification of m^6^A in total RNA extracted from pancreatic cancer cell lines and human pancreatic cancer tissues, as indicated. * *p* < 0.05; ***p < 0.001. (C) Number of m^6^A peaks and m^6^A-modified transcripts identified via m^6^A-Seq per group (T, pancreatic cancer tissue; S, adjacent tissue; N, normal pancreatic tissue). (D) Top consensus motif identified from m^6^A-Seq peaks in all tissue samples. (E) Distribution patterns of m^6^A identified via m^6^A-Seq among total and unique peaks in all groups. (F) Distribution patterns of m^6^A identified via m^6^A-Seq among the total and unique peaks in the groups, as indicated. (G) GO analysis of the m^6^A-modified transcripts unique in the groups, as indicated. (H) KEGG pathway analysis of the m^6^A-modified transcripts unique in the groups, as indicated.

**Additional file 2: Figure S2**. Protein level of METTL 3-METTL14 complex in pancreatic cancer. METTL3, METTL14 and WTAP levels in paired pancreatic cancer tissues (T) and the surrounding tissues (NT) were analyzed by western blotting.

**Additional file 3: Figure S3.** METTL14 silencing reduces pancreatic cancer cells’ proliferation and invasion. (A) Real-time PCR and validation of the efficiency of shRNA *METTL14* downregulation in PANC-1 cells. ***p < 0.001. (B) Real-time PCR showing the relative *METTL14* mRNA levels in PANC-1 cells transfected with control shRNA, shMETTL14, or shMETTL14 with shRNA-resistant METTL14. ***p < 0.001. (C) Western blot validation of the efficiency of shRNA *METTL14* downregulation and lentiviral overexpression of *METTL14* in PANC-1 cells. (D) Western blot revealing METTL14 protein expression in PANC-1 cells transfected with control shRNA, shMETTL14, or shMETTL14 with shRNA-resistant METTL14. (E) Viability of MIA PaCa-2 cells expressing shCtrl or shMETTL14, and of BxPC-3 cells stably expressing vector or *METTL14*, detected using the CCK8 assay. * *p* < 0.05; ** *p* < 0.01. (F) Viability of PANC-1 cells expressing control shRNA, shMETTL14, or shMETTL14 with shRNA-resistant METTL14. ***p < 0.001. (G) Representative images from the colony-forming assay (lower panel) and colony number analysis (upper panel). * *p* < 0.05. (H) Growth curve of subcutaneous tumors in the indicated groups; ***, p < 0.001. (I) MIA PaCa-2 cells expressing shCtrl or shMETTL14, and BxPC-3 cells stably expressing vector or *METTL14* were analyzed in a transwell assay with or without Matrigel. All experiments were performed in triplicate and data are presented as the mean ± SD. Scale bar: 200 μm. * p < 0.05; ** p < 0.01. (J) PANC-1 cells expressing control shRNA, shMETTL14, or shMETTL14 with shRNA-resistant METTL14 were analyzed in a transwell assay with or without Matrigel. ** p < 0.01; ***p < 0.001;# p < 0.01. (K) MIA PaCa-2 cells expressing shCtrl or shMETTL14 were analyzed in a wound-healing assay. All experiments were performed in triplicate and data are presented as the mean ± SD. Scale bar: 200 μm. ** p < 0.01. (L) Bodyweight curves and Kaplan-Meier analysis of the overall survival per group, as indicated, in the orthotopic transplantation mouse model. * p < 0.05; ***p < 0.001.

**Additional file 4: Figure S4.** Identification of METTL14 targets via RNA-Seq and m^6^A-Seq. (A-C) GO and KEGG pathway analysis of differentially expressed genes in PANC-1-shMETTL14 cells compared with PANC-1-shCtrl cells. (D) Number of m^6^A-modified mRNAs identified in m^6^A-seq. Common m^6^A mRNAs contain at least 1 common m^6^A peak, while unique m^6^A mRNAs contain no common m^6^A peaks. (E, F) GO and KEGG pathway analysis of m^6^A-modified transcripts in PANC-1-shMETTL14 cells compared with PANC-1-shCtrl cells.

**Additional file 5: Figure S5.** PREP is an essential METTL14 target in pancreatic cancer. (A) Relative mRNA levels of the 6 most relevant genes identified in the METTL14 downstream analysis. * p < 0.05; ** p < 0.01; ***p < 0.001; n.s., no significance. (B) Correlation analysis of *PERP* and *METTL3*, *METTL14*, and *WTAP* mRNA expression, based on a TCGA dataset of 183 pancreatic cancer patients. The gene expression profile was analyzed using the Illumina HiSeq pancan normalized pattern. Unit: pan-cancer normalized log2(norm_count+ 1). (C) Correlation analysis of *PERP* mRNA and METTL14 protein levels in the 20 pairs of specimens from this study.

**Additional file 6: Table S1.** Association between clinicopathological features and m^6^A mRNA levels.

**Additional file 7: Table S2.** m^6^A patient peak annotation.

**Additional file 8: Table S3.** shMETTL14 vs shCtrl differential expression.

**Additional file 9: Table S4.** shMETTL14 m^6^A experiment peak annotation.

**Additional file 10: Table S5.** Reagents and antibodies.

**Additional file 11: Table S6.** Samples’ information.

## Data Availability

All data generated or analyzed during this study are included in this published article (and its supplementary information files).

## Abbreviations

m^6^A: *N*^*6*^- methyladenosine
METTL14: Methyltransferase-like 14
ALKBH5: AlkB homolog 5
METTL3: Methyltransferase-like 3
FTO: Fat mass and obesity associated protein
YTHDF2: YTH domain family 2
WTAP: Wilms’ tumor 1-associating protein
HPDE: Human pancreatic duct epithelial cells
TCGA: The Cancer Genome Atlas
MeRIP: Methylated RNA immunoprecipitation
UTR: Untranslated region
PDAC: Pancreatic ductal adenocarcinoma
