# Supplementary figures and images for: Upregulation of METTL14 mediates the elevation of PERP mRNA N6 adenosine methylation promoting the growth and metastasis of pancreatic cancer
Source: Mol Cancer. 2020 Aug 25;19:130. doi: 10.1186/s12943-020-01249-8 (PMC7446161; doi:10.1186/s12943-020-01249-8)

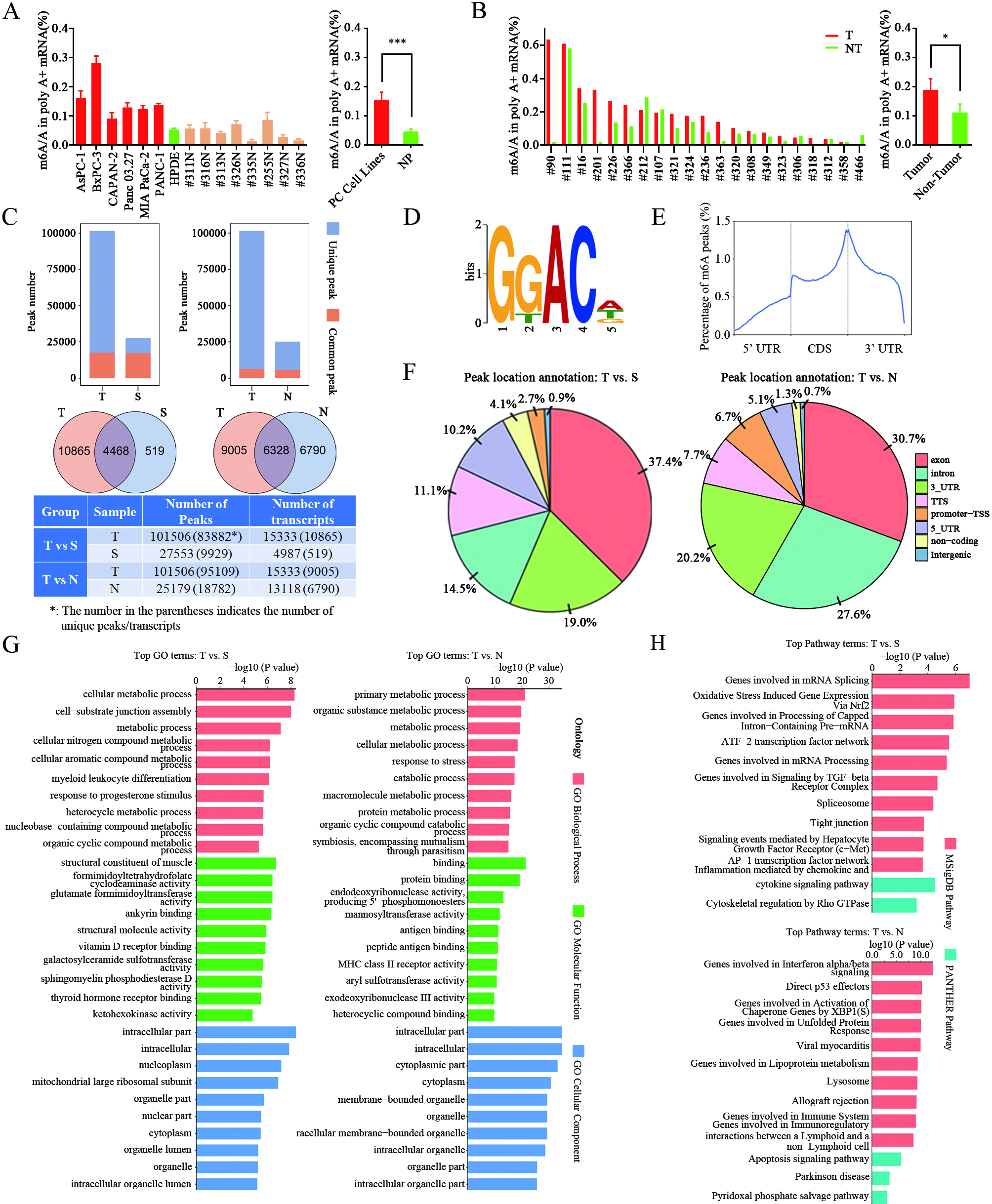

Supplement: Supplementary file 1 — Additional file 1: Figure S1. m6A modification levels and profile in pancreatic cancer. (A, B) Colorimetric quantification of m6A in total RNA extracted from pancreatic cancer cell lines and human pancreatic cancer tissues, as indicated. * p < 0.05; ***p < 0.001. (C) Number of m6A peaks and m6A-modified transcripts identified via m6A-Seq per group (T, pancreatic cancer tissue; S, adjacent tissue; N, normal pancreatic tissue). (D) Top consensus motif identified from m6A-Seq peaks in all tissue samples. (E) Distribution patterns of m6A identified via m6A-Seq among total and unique peaks in all groups. (F) Distribution patterns of m6A identified via m6A-Seq among the total and unique peaks in the groups, as indicated. (G) GO analysis of the m6A-modified transcripts unique in the groups, as indicated. (H) KEGG pathway analysis of the m6A-modified transcripts unique in the groups, as indicated. [file 12943_2020_1249_MOESM1_ESM.tif]

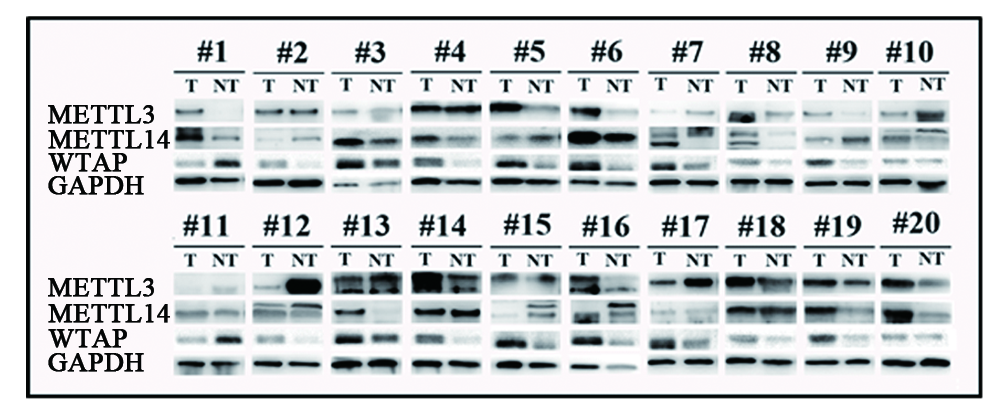

Supplement: Supplementary file 2 — Additional file 2: Figure S2. Protein level of METTL 3-METTL14 complex in pancreatic cancer. METTL3, METTL14 and WTAP levels in paired pancreatic cancer tissues (T) and the surrounding tissues (NT) were analyzed by western blotting. [file 12943_2020_1249_MOESM2_ESM.tif]

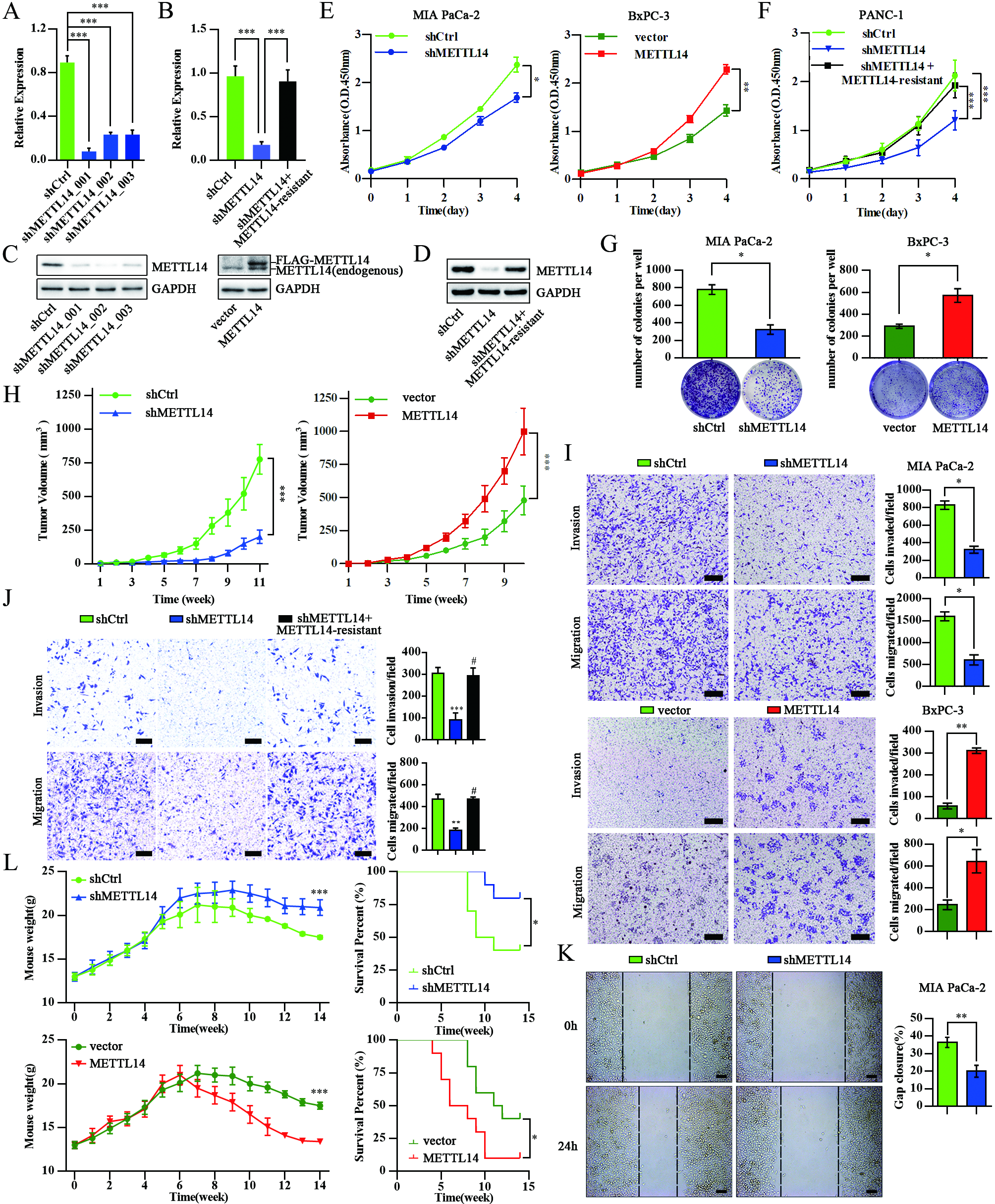

Supplement: Supplementary file 3 — Additional file 3: Figure S3. METTL14 silencing reduces pancreatic cancer cells’ proliferation and invasion. (A) Real-time PCR and validation of the efficiency of shRNA METTL14 downregulation in PANC-1 cells. ***p < 0.001. (B) Real-time PCR showing the relative METTL14 mRNA levels in PANC-1 cells transfected with control shRNA, shMETTL14, or shMETTL14 with shRNA-resistant METTL14. ***p < 0.001. (C) Western blot validation of the efficiency of shRNA METTL14 downregulation and lentiviral overexpression of METTL14 in PANC-1 cells. (D) Western blot revealing METTL14 protein expression in PANC-1 cells transfected with control shRNA, shMETTL14, or shMETTL14 with shRNA-resistant METTL14. (E) Viability of MIA PaCa-2 cells expressing shCtrl or shMETTL14, and of BxPC-3 cells stably expressing vector or METTL14, detected using the CCK8 assay. * p < 0.05; ** p < 0.01. (F) Viability of PANC-1 cells expressing control shRNA, shMETTL14, or shMETTL14 with shRNA-resistant METTL14. ***p < 0.001. (G) Representative images from the colony-forming assay (lower panel) and colony number analysis (upper panel). * p < 0.05. (H) Growth curve of subcutaneous tumors in the indicated groups; ***, p < 0.001. (I) MIA PaCa-2 cells expressing shCtrl or shMETTL14, and BxPC-3 cells stably expressing vector or METTL14 were analyzed in a transwell assay with or without Matrigel. All experiments were performed in triplicate and data are presented as the mean ± SD. Scale bar: 200 μm. * p < 0.05; ** p < 0.01. (J) PANC-1 cells expressing control shRNA, shMETTL14, or shMETTL14 with shRNA-resistant METTL14 were analyzed in a transwell assay with or without Matrigel. ** p < 0.01; ***p < 0.001;# p < 0.01. (K) MIA PaCa-2 cells expressing shCtrl or shMETTL14 were analyzed in a wound-healing assay. All experiments were performed in triplicate and data are presented as the mean ± SD. Scale bar: 200 μm. ** p < 0.01. (L) Bodyweight curves and Kaplan-Meier analysis of the overall survival per group, as indicated, in [file 12943_2020_1249_MOESM3_ESM.tif]

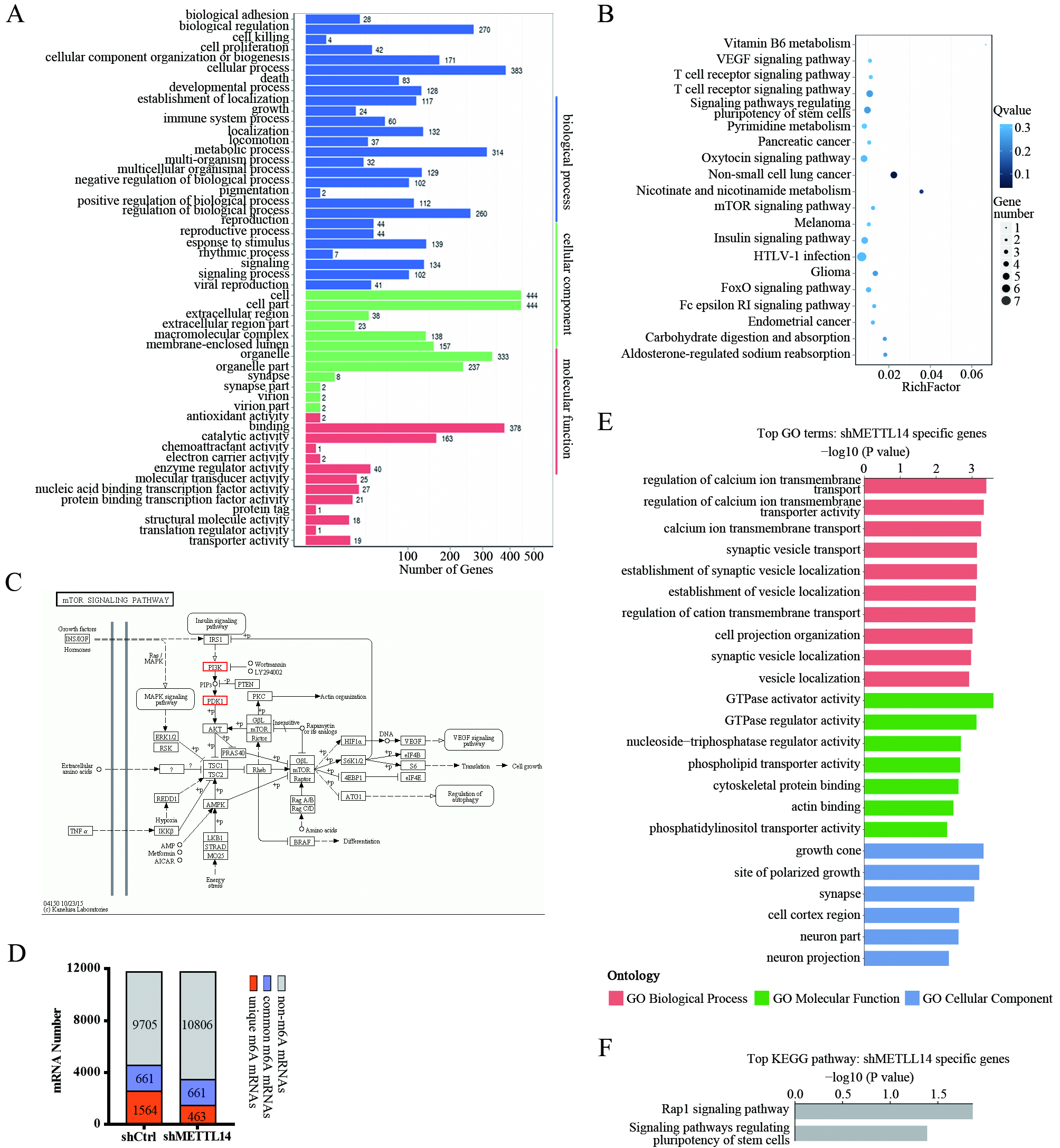

Supplement: Supplementary file 4 — Additional file 4: Figure S4. Identification of METTL14 targets via RNA-Seq and m6A-Seq. (A-C) GO and KEGG pathway analysis of differentially expressed genes in PANC-1-shMETTL14 cells compared with PANC-1-shCtrl cells. (D) Number of m6A-modified mRNAs identified in m6A-seq. Common m6A mRNAs contain at least 1 common m6A peak, while unique m6A mRNAs contain no common m6A peaks. (E, F) GO and KEGG pathway analysis of m6A-modified transcripts in PANC-1-shMETTL14 cells compared with PANC-1-shCtrl cells. [file 12943_2020_1249_MOESM4_ESM.tif]

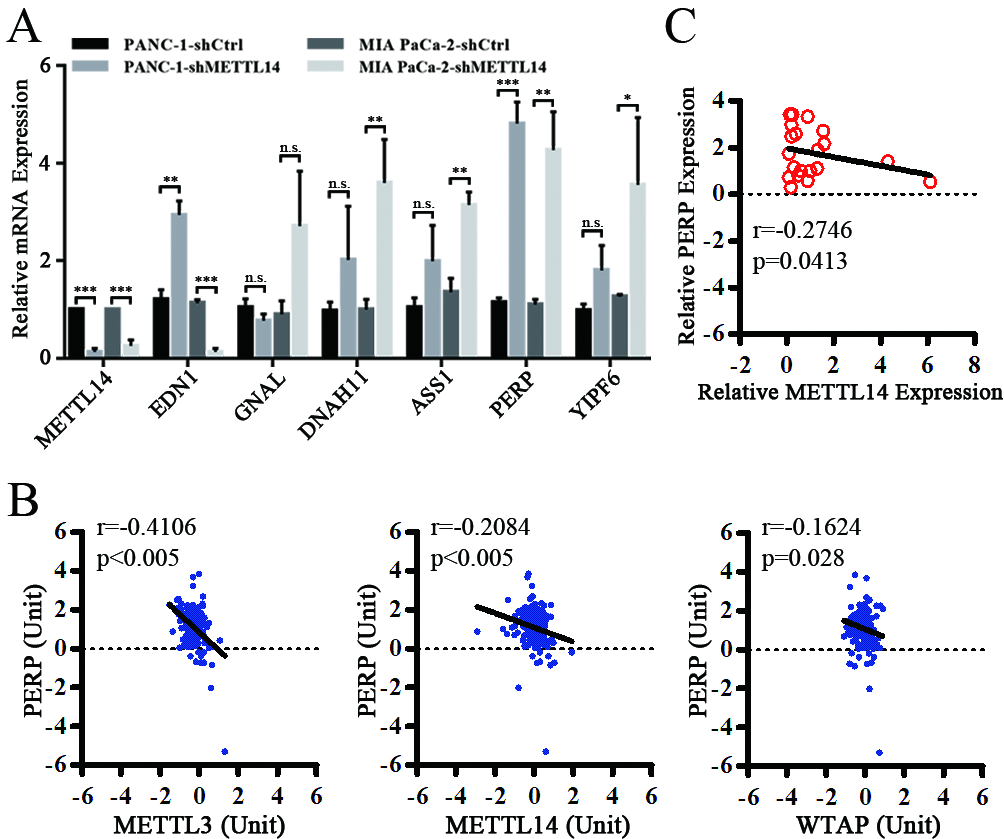

Supplement: Supplementary file 5 — Additional file 5: Figure S5. PREP is an essential METTL14 target in pancreatic cancer. (A) Relative mRNA levels of the 6 most relevant genes identified in the METTL14 downstream analysis. * p < 0.05; ** p < 0.01; ***p < 0.001; n.s., no significance. (B) Correlation analysis of PERP and METTL3, METTL14, and WTAP mRNA expression, based on a TCGA dataset of 183 pancreatic cancer patients. The gene expression profile was analyzed using the Illumina HiSeq pancan normalized pattern. Unit: pan-cancer normalized log2(norm_count+ 1). (C) Correlation analysis of PERP mRNA and METTL14 protein levels in the 20 pairs of specimens from this study. [file 12943_2020_1249_MOESM5_ESM.tif]
