# Supplementary material for: Upregulation of METTL14 mediates the elevation of PERP mRNA N6 adenosine methylation promoting the growth and metastasis of pancreatic cancer
Source: Mol Cancer. 2020 Aug 25;19:130. doi: 10.1186/s12943-020-01249-8 (PMC7446161; doi:10.1186/s12943-020-01249-8)
Supplement: Supplementary file 6 — Additional file 6: Table S1. Association between clinicopathological features and m6A mRNA levels. [file 12943_2020_1249_MOESM6_ESM.docx]

**Table S1:** **Association between clinicopathological features and m^6^A mRNA methylation level (HPLC/MS) in PC**

|  | **N** | **m^6^A mRNA methylation level** | | ***p* value** |
| --- | --- | --- | --- | --- |
|  |  | **High(11)** | **Low(10)** |  |
| Age, years |  |  |  | 1.000 |
| <50 | 9 | 5 | 4 |  |
| ≥50 | 12 | 6 | 6 |  |
| Gender |  |  |  | 0.670 |
| Female | 10 | 6 | 4 |  |
| male | 11 | 5 | 6 |  |
| Differentiation |  |  |  | 0.670 |
| Ⅰ-Ⅱ | 12 | 7 | 5 |  |
| Ⅲ-Ⅳ | 9 | 4 | 5 |  |
| Tumor size, cm |  |  |  | 0.361 |
| ≤2 | 15 | 9 | 6 |  |
| >2 | 6 | 2 | 4 |  |
| Lymph invasion |  |  |  | 0.008 |
| Yes | 13 | 10 | 3 |  |
| No | 8 | 1 | 7 |  |

The *p* value was calculated by spss with fisher's exact test.
